# Time-Fractional Diffusion with Mass Absorption in a Half-Line Domain due to Boundary Value of Concentration Varying Harmonically in Time

**DOI:** 10.3390/e20050346

**Published:** 2018-05-06

**Authors:** Yuriy Povstenko, Tamara Kyrylych

**Affiliations:** 1 Institute of Mathematics and Computer Sciences, Faculty of Mathematical and Natural Sciences, Jan Długosz University in Czȩstochowa, al. Armii Krajowej 13/15, 42-200 Czȩstochowa, Poland; 2 Institute of Law, Administration and Management, Faculty of Philology and History, Jan Długosz University in Czȩstochowa, Zbierskiego 2/4, 42-200 Czȩstochowa, Poland

**Keywords:** fractional calculus, Caputo derivative, Mittag–Leffler function, Laplace transform, sin-Fourier transform

## Abstract

The time-fractional diffusion equation with mass absorption is studied in a half-line domain under the Dirichlet boundary condition varying harmonically in time. The Caputo derivative is employed. The solution is obtained using the Laplace transform with respect to time and the sin-Fourier transform with respect to the spatial coordinate. The results of numerical calculations are illustrated graphically.

## 1. Introduction

From a mathematical point of view, diffusion and heat conduction are described by the same equation of the parabolic type

(1) $$\frac{\partial u}{\partial t}=a\Delta u$$

where *a* is the diffusivity coefficient, *t* denotes time, Δ is the Laplace operator, and *u* stands for concentration in the case of diffusion and for temperature in the case of heat conduction.

Ångström [1] was the first to consider Equation (1) under harmonic impact. In that case, sometimes the terms “oscillatory diffusion” or “diffusion waves” are used [2,3,4]. Introducing of oscillations into the diffusion equation can be done by two ways. The first possibility consists in considering the harmonic source term

(2) $$\frac{\partial u(x,t)}{\partial t}=a\frac{\partial^{2}u\left(x,t\right)}{\partial x^{2}}+f\left(x\right)\;e^{i\omega t}.$$

Nowacki [5,6] studied the equation

(3) $$\frac{\partial u(x,t)}{\partial t}=a\frac{\partial^{2}u\left(x,t\right)}{\partial x^{2}}+Q_{0}\;\delta \left(x\right)\;e^{i\omega t}$$

in the domain −∞<x<∞ with δ(x) being the Dirac delta function under assumption

(4) $$u\left(x,t\right)=U\left(x\right)\;e^{i\omega t}$$

and obtained the solution

(5) $$u\left(x,t\right)=\frac{Q_{0}}{2a\sqrt{i\omega /a}}\;\exp\left(i\omega t-\left|x\right|\sqrt{\frac{i\omega }{a}}\right).$$

The square root $\sqrt{i\omega }$ is defined as a particular case of the general formula [7]

(6) $${\left(\pm i\omega \right)}^{\alpha }={\left|\omega \right|}^{\alpha }\;e^{\pm \alpha \pi i\;\mathrm{sign}\left(\omega \right)/2}.$$

Another possibility to introduce oscillations in the diffusion equation consists in imposing the harmonic boundary condition. For example, the diffusion equation

(7) $$\frac{\partial u(x,t)}{\partial t}=a\frac{\partial^{2}u\left(x,t\right)}{\partial x^{2}}$$

is considered in the domain 0<x<∞ under condition

(8) $$x=0:\;\;u\left(x,t\right)=u_{0}\;e^{i\omega t}$$

as well as under Assumption (4). The solution has the following form:

(9) $$u\left(x,t\right)=u_{0}\;\exp\left(i\omega t-x\sqrt{\frac{i\omega }{a}}\right).$$

For the boundary condition

(10) $$x=0:\;\;u\left(x,t\right)=u_{0}\;\cos\left(\omega t\right)$$

the solution becomes [8,9]

(11) $$u\left(x,t\right)=u_{0}\;\exp\left(-x\sqrt{\frac{\omega }{2a}}\right)\cos\left(\omega t-x\sqrt{\frac{\omega }{2a}}\right),$$

and for the boundary condition

(12) $$x=0:\;\;u\left(x,t\right)=u_{0}\;\sin\left(\omega t\right)$$

one obtains [10]

(13) $$u\left(x,t\right)=u_{0}\;\exp\left(-x\sqrt{\frac{\omega }{2a}}\right)\sin\left(\omega t-x\sqrt{\frac{\omega }{2a}}\right).$$

In a medium with a chemical reaction or with heat absorption/release, in Equation (1) , there appears an additional linear term [11,12]

(14) $$\frac{\partial u}{\partial t}=a\Delta u-bu.$$

The values of the coefficient b>0 and b<0 correspond to mass/heat absorption and mass/heat release, respectively. Equation (14) also describes mass or heat transport in a thin plate which lateral surfaces exchange mass or heat with surroundings [13] as well as bio-heat transfer [14,15,16]. In the case of one spatial variable, Equation (14) governs propagation of neuronal signals and is known as the cable equation [17,18].

The hyperbolic Klein–Gordon equation is useful in different physical theories, for example, in solid state physics, quantum field theory, classical mechanics, and nonlinear optics [19,20]:

(15) $$\frac{\partial^{2}u}{\partial t^{2}}=a\Delta u-bu.$$

In materials with complex internal structure (amorphous, porous, random and disordered media, polymers, glasses, dielectrics, and semiconductors) memory effects play an important role [21,22]. The “long-tail” memory with power kernel can be interpreted in terms of fractional calculus. The theory of integrals and derivatives of non-integer order has many applications in physics, chemistry, biology, and engineering (see [23,24,25,26,27,28,29,30,31,32,33] and references therein).

The time-fractional counterpart of Equation (1) has the form [34,35,36]

(16) $$\frac{\partial^{\alpha }u}{\partial t^{\alpha }}=a\Delta u,\;0<\alpha \leq 2$$

where $\partial^{\alpha }u\left(x,t\right)/\partial t^{\alpha }$ is the left-sided Caputo fractional derivative [7,23]

(17) $$\frac{\partial^{\alpha }u\left(x,t\right)}{\partial t^{\alpha }}=\frac{1}{\Gamma (n-\alpha )}\int_{0}^{t}{\left(t-\tau \right)}^{n-\alpha -1}\frac{\partial^{n}u\left(x,\tau \right)}{\partial \tau^{n}}\;d\tau ,\;n-1<\alpha <n$$

with Γ(α) being the gamma function. The Caputo derivative has the Laplace transform rule

(18) $$L\left\{\frac{d^{\alpha }f\left(t\right)}{dt^{\alpha }}\right\}=s^{\alpha }f^{*}\left(s\right)-\sum_{k=0}^{n-1}f^{\left(k\right)}\left(0^{+}\right)s^{\alpha -1-k},\;n-1<\alpha <n.$$

Here, the asterisk denotes the transform, and *s* is the Laplace transform variable.

The equation

(19) $$\frac{\partial^{\alpha }u}{\partial t^{\alpha }}=a\Delta u-bu,\;0<\alpha \leq 2$$

can be regarded as the time-fractional generalization of the diffusion, bio-heat, and cable equations as well as time-fractional generalization of the Klein–Gordon equation [37,38,39,40,41,42,43].

In this paper, we study Equation (19) in a half-line domain under the Dirichlet boundary condition varying harmonically in time; the paper develops the results of [44].

## 2. The Statement and Solution of the Problem

The equation

(20) $$\frac{\partial^{\alpha }u\left(x,t\right)}{\partial t^{\alpha }}=a\frac{\partial^{2}u\left(x,t\right)}{\partial x^{2}}-bu\left(x,t\right),\;0<\alpha \leq 2$$

is considered in the domain 0<x<∞ under the boundary condition

(21) $$x=0:\;\;u\left(x,t\right)=u_{0}\;e^{i\omega t}.$$

For later use in numerical calculations, it is appropriate to introduce the following nondimensional quantities:

(22) $$\bar{u}=\frac{u}{u_{0}},\;\bar{x}=\frac{x}{\sqrt{a}t^{\alpha /2}},\;\bar{b}=bt^{\alpha }.\;\bar{\omega }=\omega t.$$

First, we investigate two particular cases of Equation (20) corresponding to the integer values of the order α under Assumption (4).

### 2.1. Bio-Heat Equation: Quasi-Steady-State Oscillations

In this case α=1 and for the function U(x) we have equations

(23) $$a\;\frac{d^{2}U\left(x\right)}{dx^{2}}=\left(b+i\omega \right)U\left(x\right)$$

and

(24) $$x=0:\;\;U\left(x\right)=u_{0}.$$

Using the sin-Fourier integral transform, we obtain

(25) $$\tilde{U}\left(\xi \right)=\frac{au_{0}\xi }{a\xi^{2}+b+i\omega }$$

where the tilde denotes the transform, and ξ is the transform variable. The inverse sin-Fourier transform yields for b>0

(26) $$U\left(x\right)=u_{0}\exp\left(-x\sqrt{\frac{b+i\omega }{a}}\right)$$

and

(27) $$u\left(x,t\right)=u_{0}\exp\left(i\omega t-x\sqrt{\frac{b+i\omega }{a}}\right).$$

If the boundary condition is

(28) $$x=0:\;\;u\left(x,t\right)=u_{0}\cos\left(\omega t\right),$$

then

(29) $$u\left(x,t\right)=u_{0}\exp\left[-\frac{x\sqrt{r}}{\sqrt{a}}\cos\left(\frac{\varphi }{2}\right)\right]\cos\left[\omega t-\frac{x\sqrt{r}}{\sqrt{a}}\sin\left(\frac{\varphi }{2}\right)\right]$$

where

(30) $$r=\sqrt{b^{2}+\omega^{2}},\;\varphi =\arctan\left(\frac{\omega }{b}\right).$$

**Figure 1:**
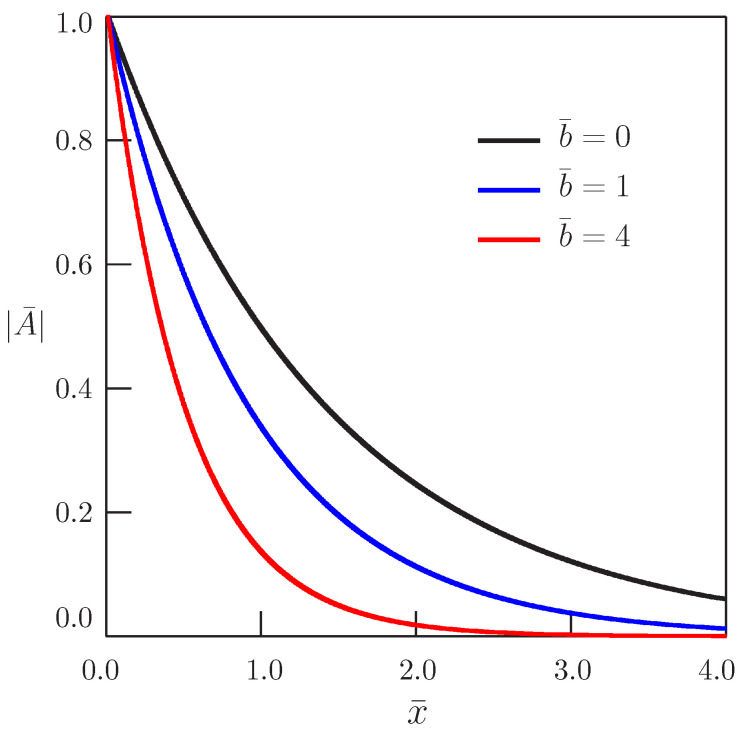
Dependence of the oscillation amplitude of the solution in Equation (29) on distance for the bio-heat equation (α=1, $\bar{\omega }=1$).

Figure 1 shows the dependence of the amplitude of oscillations described by Equation (29) on distance for different values of the parameter *b*.

### 2.2. Klein–Gordon Equation: Quasi-Steady State Oscillations

For the hyperbolic Klein–Gordon equation (α=2)

(31) $$\frac{\partial^{2}u\left(x,t\right)}{\partial t^{2}}=a\frac{\partial^{2}u\left(x,t\right)}{\partial x^{2}}-bu\left(x,t\right)$$

under the boundary condition

(32) $$x=0:\;\;u\left(x,t\right)=u_{0}\;e^{i\omega t}$$

and Assumption (4), we obtain

(33) $$a\;\frac{d^{2}U\left(x\right)}{dx^{2}}=\left(b-\omega^{2}\right)U\left(x\right)$$

and

(34) $$x=0:\;\;U\left(x\right)=u_{0}.$$

Applying the sin-Fourier transform to Equation (33), we obtain

(35) $$\tilde{U}\left(\xi \right)=\frac{u_{0}a\xi }{a\xi^{2}+b-\omega^{2}}.$$

The inversion of the transform depends on the relation between *b* and ω. For $b>\omega^{2}$

(36) $$u\left(x,t\right)=u_{0}\exp\left(-x\sqrt{\frac{b-\omega^{2}}{a}}+i\omega t\right),$$

whereas, for $b<\omega^{2}$,

(37) $$u\left(x,t\right)=u_{0}\cos\left(-x\sqrt{\frac{\omega^{2}-b}{a}}\right)e^{i\omega t}.$$

**Figure 2:**
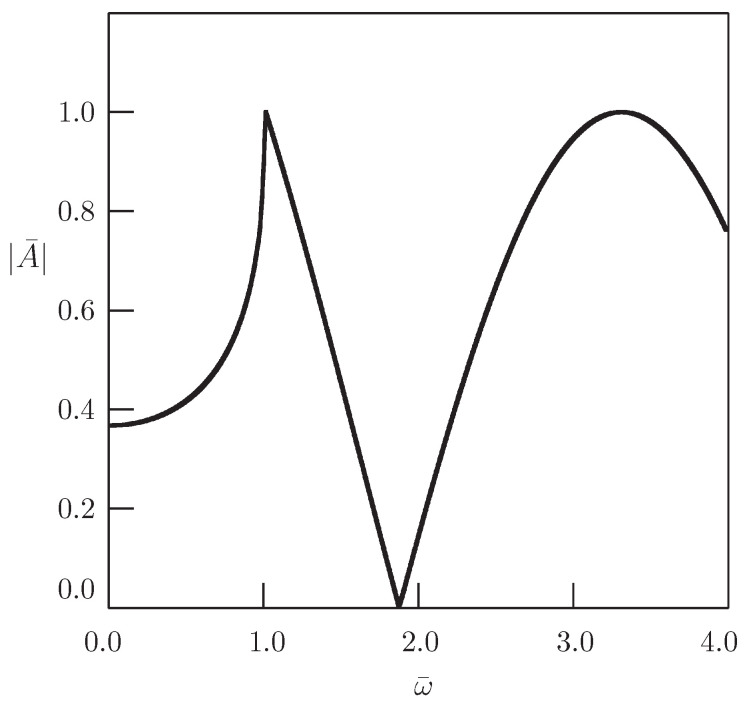
Dependence of the amplitude of oscillations governed by the solution in Equations (36) and (37) on the frequency for the Klein–Gordon equation in the domain 0<x<∞ with the harmonic Dirichlet boundary condition (α=2, $\bar{b}=1$, $\bar{x}=1$).

Figure 2 shows the dependence of the nondimensional amplitude of oscillations described by Equations (36) and (37) on the frequency ω for $\bar{x}=1$.

**Figure 3:**
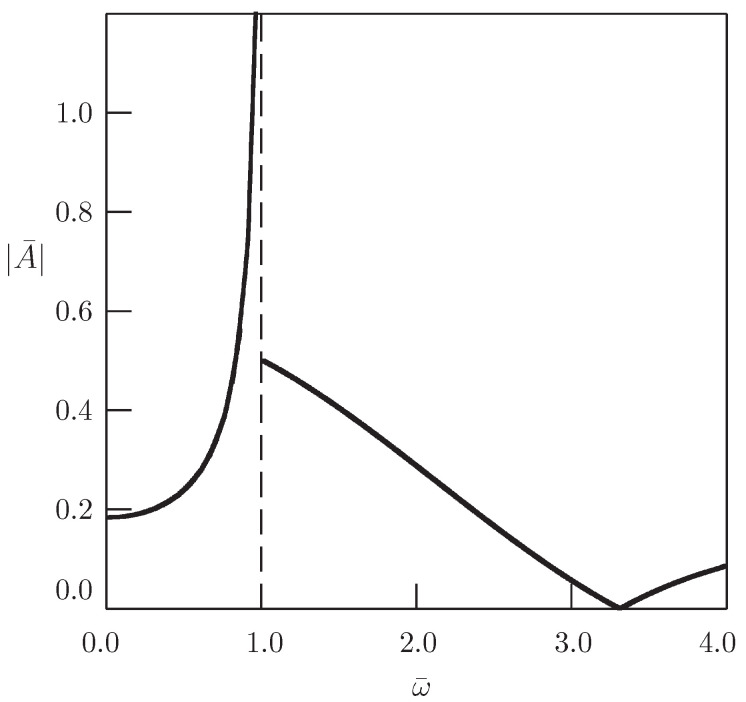
Dependence of the oscillation amplitude on the frequency for the Klein–Gordon equation in the domain −∞<x<∞ with the harmonic source term (α=2, $\bar{b}=1$, $\bar{x}=1$); see [44].

It should be noted the difference between the result of the present paper and the corresponding result of [44]. If Equation (31) is considered with the harmonic source term $\delta \left(x\right)\;e^{i\omega t}$ in the domain −∞<x<∞ under Assumption (4), then there appears the resonance at $\omega =\sqrt{b}$ (see Figure 3). In the case of the half-line domain with the harmonic Dirichlet boundary condition, there is no resonance.

### 2.3. Equation with Time-Fractional Derivative

It should be emphasized that the equation with time-fractional derivative

(38) $$\frac{\partial^{\alpha }u\left(x,t\right)}{\partial t^{\alpha }}=a\frac{\partial^{2}u\left(x,t\right)}{\partial x^{2}}-bu\left(x,t\right),\;0<\alpha \leq 2$$

under the boundary condition

(39) $$x=0:\;\;u\left(x,t\right)=u_{0}\;e^{i\omega t}$$

cannot be considered under Assumption (4). This is due to the formula [45]

(40) $$\frac{d^{\alpha }e^{\lambda t}}{dt^{\alpha }}=\lambda^{\alpha }e^{\lambda t}\;\frac{\gamma (n-\alpha ,\lambda t)}{\Gamma (n-\alpha )}\neq \lambda^{\alpha }e^{\lambda t},\;n-1<\alpha <n$$

where γ(a,x) is the incomplete gamma function [46]

(41) $$\gamma \left(a,x\right)=\int_{0}^{x}e^{-w}\;w^{a-1}\;dw.$$

For equations with time-derivative of the fractional order, the initial conditions should be imposed; for example,

(42) t=0:u(x,t)=0,0<α≤1,

and

(43) $$t=0:\;\;\frac{\partial u(x,t)}{\partial t}=0,\;1<\alpha \leq 2.$$

The Laplace transform with respect to time *t* and the sin-Fourier transform with respect to the spatial coordinate *x* yield

(44) $${\tilde{u}}^{*}\left(\xi ,s\right)=\frac{u_{0}a\xi }{s^{\alpha }+a\xi^{2}+b}\;\frac{1}{s-i\omega }.$$

Inversion of the integral transform using the convolution theorem results in the solution

(45) $$u\left(x,t\right)=\frac{2au_{0}}{\pi }\;\int_{0}^{\infty }\int_{0}^{t}\tau^{\alpha -1}E_{\alpha ,\alpha }\left[-\left(a\xi^{2}+b\right)\tau^{\alpha }\right]e^{i\omega (t-\tau )}\;\xi \;\sin\left(x\xi \right)d\tau \;d\xi$$

where the following formula [7,23]

(46) $$L^{-1}\left\{\frac{s^{\alpha -\beta }}{s^{\alpha }+p}\right\}=t^{\beta -1}E_{\alpha ,\beta }\left(-pt^{\alpha }\right)$$

has been used. Here $E_{\alpha ,\beta }\left(z\right)$ is the Mittag–Leffler function in two parameters α and β:

(47) $$E_{\alpha ,\beta }\left(z\right)=\sum_{n=0}^{\infty }\frac{z^{n}}{\Gamma (\alpha n+\beta )},\;\alpha >0,\;\beta >0,\;z\in C.$$

### 2.4. Bio-Heat Equation: The Solution with Zero Initial Condition

Equation (44) for α=1 takes the form

(48) $${\tilde{u}}^{*}\left(\xi ,s\right)=\frac{u_{0}a\xi }{s+a\xi^{2}+b}\;\frac{1}{s-i\omega }=\frac{u_{0}a\xi }{a\xi^{2}+b+i\omega }\left(\frac{1}{s-i\omega }-\frac{1}{s+a\xi^{2}+b}\right).$$

The inverse Laplace transform results in

(49) $$\tilde{u}\left(\xi ,t\right)=\frac{u_{0}a\xi }{a\xi^{2}+b+i\omega }\left[e^{i\omega t}-e^{-\left(a\xi^{2}+b\right)t}\right]$$

and finally

(50) $$\begin{array}{cc} u(x,t) & =u_{0}\;\exp\left(i\omega t-x\sqrt{\frac{b+i\omega }{a}}\right)\\ & -\frac{u_{0}}{2}\;\exp\left(i\omega t-x\sqrt{\frac{b+i\omega }{a}}\right)\;\mathrm{erfc}\left[\sqrt{(b+i\omega )t}-\frac{x}{2\sqrt{at}}\right]\\ & +\frac{u_{0}}{2}\;\exp\left(i\omega t+x\sqrt{\frac{b+i\omega }{a}}\right)\;\mathrm{erfc}\left[\sqrt{(b+i\omega )t}+\frac{x}{2\sqrt{at}}\right], \end{array}$$

where erfc(x) is the complementary error function.

### 2.5. Klein–Gordon Equation: The Solution with Zero Initial Conditions

For α=2, Equation (44) becomes

(51) $${\tilde{u}}^{*}=\frac{u_{0}a\xi }{s^{2}+a\xi^{2}+b}\;\frac{1}{s-i\omega }.$$

The inverse sin-Fourier transform yields

(52) $$u^{*}\left(x,s\right)\frac{u_{0}}{s-i\omega }\;\exp\left(-\frac{x}{\sqrt{a}}\;\sqrt{s^{2}+b}\right).$$

The inverse Laplace transform of $\exp\left(-x\sqrt{s^{2}+b}/\sqrt{a}\right)$ depends on the sign of *b* and reads [47]:

(i) For b>0,
  (53) $$L^{-1}\left\{\exp\left(-\frac{x}{\sqrt{a}}\;\sqrt{s^{2}+b}\right)\right\}=\left\{\begin{array}{cc} \;\;\delta \left(t-x/\sqrt{a}\right)-\frac{x\sqrt{b}}{\sqrt{a}}\;\frac{J_{1}\left(\sqrt{b}\sqrt{t^{2}-x^{2}/a}\right)}{\sqrt{t^{2}-x^{2}/a}}, & x<\sqrt{a}t,\\ \;\;0, & \sqrt{a}t<x \end{array}.\right.$$
(ii) For b<0,
  (54) $$L^{-1}\left\{\exp\left(-\frac{x}{\sqrt{a}}\;\sqrt{s^{2}-\left|b\right|}\right)\right\}=\left\{\begin{array}{cc} \;\;\delta \left(t-x/\sqrt{a}\right)+\frac{x\sqrt{\left|b\right|}}{\sqrt{a}}\;\frac{I_{1}\left(\sqrt{\left|b\right|}\sqrt{t^{2}-x^{2}/a}\right)}{\sqrt{t^{2}-x^{2}/a}}, & x<\sqrt{a}t,\\ \;\;0, & \sqrt{a}t<x \end{array}.\right.$$

Here, $J_{1}\left(z\right)$ is the Bessel function of the first kind, $I_{1}\left(z\right)$ is the modified Bessel function of the first kind.

Using the convolution theorem, we obtain, for b>0,

(55) $$u\left(x,t\right)=\left\{\begin{array}{c} \;\;u_{0}\;e^{i\omega (t-x/\sqrt{a})}-u_{0}x\sqrt{b/a}\int_{x/\sqrt{a}}^{t}e^{i\omega (t-\tau )}\;\frac{J_{1}\left(\sqrt{b/a}\sqrt{a\tau^{2}-x^{2}}\right)}{\sqrt{\left(a\tau^{2}-x^{2}\right)/a}}\;d\tau ,\;\;0\leq x\sqrt{a}t\\ \;\;0,\;\sqrt{a}t<x<\infty \end{array},\right.$$

and, for b<0,

(56) $$u\left(x,t\right)=\left\{\begin{array}{c} \;\;u_{0}\;e^{i\omega (t-x/\sqrt{a})}+u_{0}x\sqrt{|b|/a}\int_{x/\sqrt{a}}^{t}e^{i\omega (t-\tau )}\;\frac{I_{1}\left(\sqrt{|b|/a}\sqrt{a\tau^{2}-x^{2}}\right)}{\sqrt{\left(a\tau^{2}-x^{2}\right)/a}}\;d\tau ,\;\;0\leq x\sqrt{a}t\\ \;\;0,\;\sqrt{a}t<x<\infty \end{array}.\right.$$

**Figure 4:**
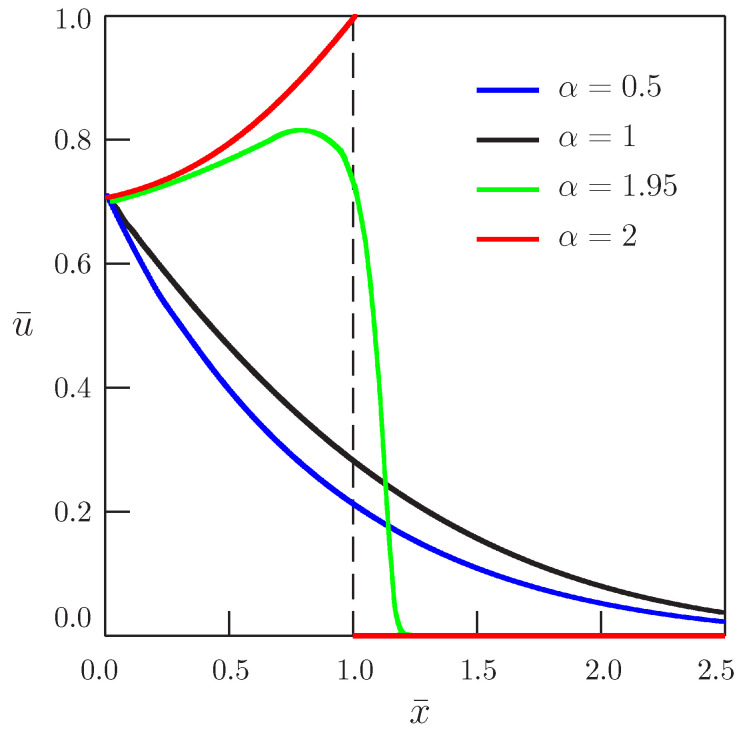
Dependence of the solution of Equation (45) on distance ($\bar{b}=1$, $\bar{\omega }=\pi /4$).

**Figure 5:**
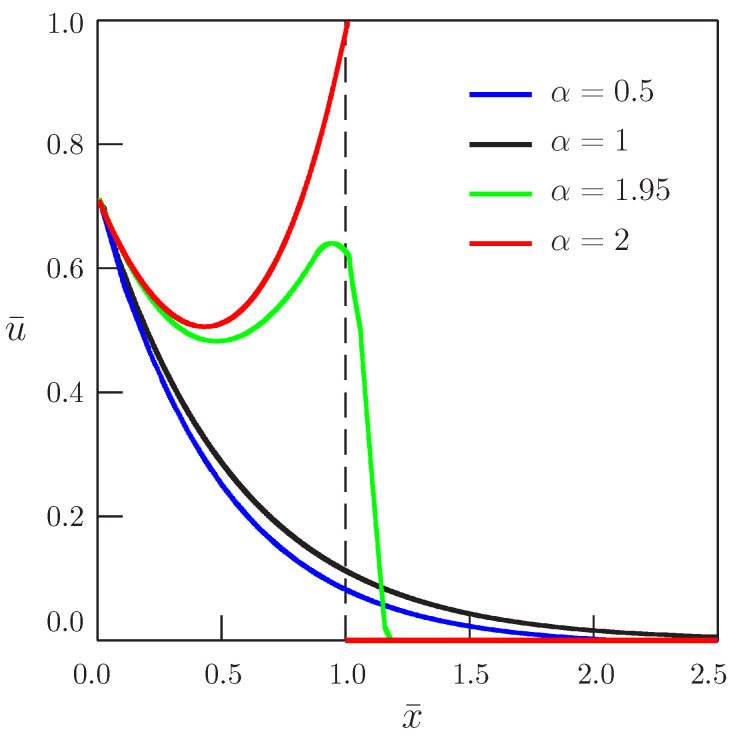
Dependence of the solution of Equation (45) on distance ($\bar{b}=4$, $\bar{\omega }=\pi /4$).

Figure 4 and Figure 5 present the dependence of the solution on the distance for $\bar{b}=1$ and $\bar{b}=4$, respectively, and for different values of the order of time-derivative α.

## 3. Conclusions

We have considered the time-fractional diffusion-wave equation with the Caputo fractional derivative of the order 0<α≤2 with mass absorption in a half-line domain under the Dirichlet boundary condition varying harmonically in time. The investigated equation can also be regarded as the time-fractional generalization of the bio-heat and Klein–Gordon equations. The Caputo derivative of the exponential function has more complicated form than the derivative of the integer order. As a consequence, the assumption that the solution can be represented as a product of a function of the spatial coordinate and the time-harmonic term without taking into account the initial conditions cannot be used. In the case of the standard Klein–Gordon equation, the solution of Equations (36) and (37) describes the quasi-steady-state oscillations, whereas the solution of Equations (55) and (56) also describes the transient process and has the wave front at $x=\sqrt{a}t$. When α approaches 2, the solution approximates this wave front.
